# The pediatric AI readiness framework: bridging evidence to practice in pediatric artificial intelligence

**DOI:** 10.3389/frai.2026.1800047

**Published:** 2026-05-01

**Authors:** Emre Sezgin, Samantha Boch

**Affiliations:** 1 Center for Biobehavioral Health, The Abigail Wexner Research Institute at Nationwide Children’s Hospital, Columbus, OH, United States; 2 Department of Pediatrics, The Ohio State University College of Medicine, Columbus, OH, United States; 3 Center for Child Health Equity and Outcomes Research, The Abigail Wexner Research Institute at Nationwide Children’s Hospital, Columbus, OH, United States; 4 Center for Nursing Excellence, Nationwide Children’s Hospital, Columbus, OH, United States

**Keywords:** artificial intelligence, children, implementation framework, pediatrics, readiness, review

## Abstract

Artificial intelligence (AI) presents a transformative opportunity for pediatric healthcare, promising advancements in early diagnosis, personalized treatment, and operational efficiency. However, the unique developmental, physiological, and ethical considerations inherent to children necessitate a specialized approach to AI integration. This study introduces the Pediatric AI Readiness (PAIR) Framework—a pediatric-focused implementation-readiness checklist intended to support clinicians, health system leaders, regulators, and developers as they plan, evaluate, and operationalize AI tools in pediatric settings. PAIR complements existing AI development, evaluation, and reporting guidance by focusing on institutional and workflow readiness for real-world use. It organizes readiness considerations across seven domains: ethics and governance, population and data representativeness, validation and testing, workflow integration, economic and sustainability analysis, low-resource adaptability, and transparent reporting. By prioritizing child-centric design, rigorous validation, and collaborative governance, the PAIR framework aims to bridge the critical gap between AI innovation and its responsible application, fostering improved design and healthier futures for children.

## 1 Introduction

Artificial intelligence (AI) has moved from a concept to a clinical reality in pediatric medicine. Over the past 5 years, research on AI applications in pediatric healthcare has surged, with a dramatic increase in scientific literature. It has demonstrated both the scale of scientific enthusiasm and the breadth of potential impact (Park et al., 2025; Bhargava et al., 2024). AI systems excel at discerning multidimensional patterns that often elude human cognition (e.g., clinicians), promising earlier diagnosis, personalized therapeutics, and population-level surveillance tailored to children’s unique developmental trajectories (Ilić and Sarajlija, 2025; Huang and Shu, 2025). However, pediatric patients are not simply “small adults.” Physiological maturation, dynamic growth, and evolving psychosocial contexts render algorithms trained on adult data unreliable (and potentially harmful) when indiscriminately applied to younger cohorts and across diverse settings (Institute of Medicine (US), 2000). Ethical and regulatory concerns likewise intensify: Minors cannot legally provide consent; rather, they provide assent in addition to the legally required parental consent; their data are inherently more sensitive, protected, and subject to heightened privacy regulations; and algorithmic bias may disproportionately disadvantage children from underrepresented groups (Chng et al., 2025; Boch et al., 2022; de Vere Hunt et al., 2025). As a result, national and international frameworks are converging to ensure that AI in pediatrics is not only technologically sound but also safe, equitable, transparent, and focused on the best interests of the child and family.

In this study, we propose the Pediatric AI Implementation Readiness (PAIR) framework to help bridge the gap between rapidly advancing AI innovations and their safe, effective, and equitable deployment in pediatric clinical settings. The PAIR is designed for stakeholders who are considering real-world implementation or scale-up of pediatric AI tools (e.g., decision support, triage, documentation, and patient-facing applications). It does not replace methodological guidance for model development or established reporting standards; rather, it translates child-specific ethical, governance, validation, workflow, and sustainability considerations into actionable readiness checkpoints. This study first synthesizes current evidence and guidance, delineates the distinct challenges that impede implementation, and then presents the PAIR along with a companion self-appraisal checklist to support practical planning and evaluation.

## 2 Clinical applications of AI in pediatrics

### 2.1 Early detection and predictive diagnosis

AI is currently used to identify pediatric diseases such as asthma (Yu et al., 2020; Steinzor, 2024), congenital heart defects (Holt et al., 2025), and autism spectrum disorders (Jia et al., 2025) at considerably earlier stages than previously possible. Predictive analytics are instrumental in this process, analyzing extensive datasets to discern subtle indicators before the symptoms develop or intensify (Macias et al., 2023). Within critical early developmental stages of childhood and in settings such as neonatal intensive care units (NICUs), machine learning algorithms continuously monitor real-time vital signs, alerting clinicians to critical conditions such as sepsis or respiratory distress prior to detection (Sullivan et al., 2023; Schouten et al., 2024). Moreover, these models are capable of diagnostic pattern recognition across intricate datasets. AI-powered tools synthesize electronic health records with genetic and imaging data to uncover the latent signatures of rare diseases (Garcelon et al., 2020; Germain et al., 2025; Chen et al., 2025). These studies and implementations emphasize the need for structured frameworks to ensure the responsible deployment of pediatric AI solutions in future iterations.

### 2.2 Personalized treatment plans and precision medicine

Acknowledging the differential responses of children to therapeutic interventions, AI models are pivotal to customizing treatment strategies for individual pediatric patients. This personalization encompasses optimizing medication dosages, therapy schedules, and post-discharge care, drawing insights from a child’s genetic markers and comprehensive medical history (Bhargava et al., 2024; Rowe and Lester, 2020; Vial and Almon, 2023; Thotapalli et al., 2025; Colakoglu et al., 2025). Furthermore, the emerging concept of digital twins in pediatric healthcare (virtual replicas of individual patients) allows for the simulation of various treatment scenarios and virtual clinical trials (Pammi et al., 2025). This enables clinicians to test interventions virtually before applying them to the actual patient, transforming personalized care and risk mitigation (Abd Elaziz et al., 2024). Such precision-based plans endeavor to curtail empirical adjustments in pediatric pharmacology, mitigate adverse effects, and expedite treatment and recovery (Crisafulli et al., 2024).

### 2.3 Operational efficiency and equitable access to care

AI tools streamline administrative responsibilities such as note-taking, prescription formulation, and follow-up scheduling, thereby enabling clinicians to dedicate more attention to patient care (Knight et al., 2023; Alboksmaty et al., 2025; Sezgin et al., 2024; Bhuyan et al., 2025). Currently, robotics-assisted autonomous procedures facilitate greater precision and shorter recovery periods for pediatric surgeries (Tsai et al., 2024). AI-enabled telemedicine is extending access to rural and underserved regions, permitting remote diagnosis, monitoring, and specialist consultations (Shah and Badawy, 2021). AI-powered virtual assistants support clinical teams in medication and symptom follow-ups and triaging (Da’Costa et al., 2025; Alqaraleh et al., 2025; Sezgin et al., 2022), concurrently assisting clinicians with decision-making tools using augmented patient data (Taylor et al., 2025). These technologies are demonstrating significant utility in low- and middle-income countries (LMICs), where AI-based platforms are scalable and capable of offering personalized guidance and escalating high-risk cases to healthcare providers (Khan et al., 2024; Ciecierski-Holmes et al., 2022).

Across these use cases, pediatric AI systems frequently interact with evolving physiology, family-centered decision making, and fragmented care environments (home, school, community, and hospital). These characteristics heighten the importance of (i) representative pediatric data and age-stratified performance reporting, (ii) validation that captures clinical utility and potential harms, and (iii) workflow integration that preserves clinician oversight and adolescent confidentiality.

## 3 Ethical pillars and governance ecosystem for pediatric AI

Pediatric AI governance is currently assembled from overlapping layers of authority. Binding statutes and enforcement define baseline privacy and consumer protection obligations [e.g., the Health Insurance Portability and Accountability Act (HIPAA) for covered entities and Children’s Online Privacy Protection Act (COPPA) for child-directed online services], while product regulation applies more narrowly when an AI tool qualifies as a medical device [e.g., under the Food and Drug Administration (FDA)]. Standards bodies [e.g., the National Institute of Standards and Technology (NIST)] and international organizations [e.g., United Nations Children’s Fund (UNICEF) and World Health Organization (WHO)] provide non-binding but influential risk management and rights-based guidance. Professional societies [e.g., the American Academy of Pediatrics (AAP) and American Medical Association (AMA)] similarly shape clinical norms and procurement expectations even when their policies are not legally binding. Together, these instruments form a layered but fragmented governance environment. What remains underdeveloped is a child-specific framework that translates these principles into operational criteria for real-world pediatric AI deployment.

### 3.1 Consent, assent, and professional oversight

Because minors lack legal decisional capacity, parental permission is often required for clinical services, while developmentally appropriate assent should be sought when feasible. For pediatric AI, the consent question differs by context: retrospective secondary use of de-identified data for model development is typically governed through institutional review, data-use agreements, and applicable privacy law, whereas AI-enabled clinical services and patient-facing tools require clear communication to families about when AI is used, what it is intended to do, and how clinicians remain accountable for decisions. The American Academy of Pediatrics (AAP) advocates for plain-language communication and developmentally appropriate assent from older children, consistent with the Plain Language Act (American Academy of Pediatrics, 2021). For adolescents, implementation must also account for evolving autonomy and confidentiality (Moon, 2012; Murdoch et al., 2023). Practical approaches include electronic health records (EHR)-embedded consent documentation and workflows that support adolescent privacy where legally permitted (Webber et al., 2019). Professional oversight remains essential. The American Medical Association frames AI as “augmented intelligence” and emphasizes that clinical AI should be used under the supervision of licensed clinicians, with the ability to override or disregard algorithmic outputs (Crigger et al., 2022). Accordingly, pediatric AI programs should establish governance processes (e.g., algorithm review committees) that include clinical, data science, ethics, and family or community perspectives to review intended use, consent communication, and accountability when errors occur (Vaccaro et al., 2024; Gorski and Rudloff, 2023).

### 3.2 Data protection and cybersecurity

Children’s Online Privacy Protection Act (COPPA) and Health Insurance Portability and Accountability Act (HIPAA) can confer heightened protection on children’s data (Federal Trade Commission, 2025; Edemekong et al., 2025), but AI introduces a new surface area for breach, misuse, and inference. Pediatric records are also uniquely diffuse, spanning parental reports, school-based screenings, wearable or remote monitoring streams, and multi-institutional EHR fragments, which heightens the risk of linkage errors or inadvertent re-identification if provenance is not meticulously logged. When an AI function meets the legal definition of a medical device (typically because it is intended to diagnose, treat, mitigate, or prevent disease), FDA oversight may apply, and such tools may be regulated as software functions (including SaMD), with expectations related to quality systems, cybersecurity, and post-market management (U.S. Food and Drug Administration, 2025; U.S. Food and Drug Administration, 2025). However, the majority of AI-enabled tools used in pediatric care (e.g., administrative optimization, documentation assistance, and some clinical decision support functions) may fall outside FDA jurisdiction or be regulated through other mechanisms, reinforcing that institutional governance, contractual controls, and security engineering remain essential regardless of regulatory classification. The NIST further supplements this effort with the AI Risk Framework to manage AI risks at AI lifecycle stages (NIST, 2021). Academic initiatives echo these works, as one of the recent frameworks for safe AI pediatric practices, ACCEPT-AI, suggests explicit data-provenance statements and end-to-end encryption for remotely collected vital signs (Muralidharan et al., 2023).

### 3.3 Bias, fairness, and equity

Age-related algorithmic bias, the systematic error that arises when adult models are transposed to children, has become a watchword in pediatric informatics (Chu et al., 2023). Applying AI models designed for adults to pediatric patients can lead to inaccurate algorithm performance, potentially causing bias or misclassification (Alkhulaifat et al., 2023). Although best practice and emerging standards for trustworthy AI call for training, testing, and validating models on pediatric cohorts representative of the intended clinical context, a recent review found that only 19% of cleared pediatric devices actually used pediatric training data (Brewster et al., 2025). However, we acknowledge that pediatric data can be scarce, potentially making implementation infeasible (de Vere Hunt et al., 2025), such as in pediatric imaging, where data account for < 1% of public medical imaging datasets (Sullivan et al., 2023). In these contexts, the ethical priority shifts from ensuring “fair deployment” of existing tools to collecting the necessary data and building the governance infrastructure necessary for fair and equitable pediatric AI development (Cheung et al., 2025). Nonetheless, ethical obligations of respect for persons, beneficence, and justice require proactive steps to assess and mitigate inequities before and after deployment (Boch et al., 2022). Current frameworks attempt to pave the way: ACCEPT-AI details pediatric data collection and model development pipeline (Muralidharan et al., 2023), PEARL-AI incorporates elements to guide safe, fair, and equitable pediatric AI implementation (Chng et al., 2025), NAM proposes the AI code of conduct framework to promote responsible and equitable AI practices (The Learning Health System Series, National Academy of Medicine, 2025), and the AMA instructs physicians to collaborate with developers and deployers from planning phase to deployment and monitoring (Crigger et al., 2022).

### 3.4 Explainability and transparency

Explainability refers to the extent to which an AI system can provide human-understandable reasons for its outputs, enabling users to interrogate recommendations and calibrate trust appropriately (Tjoa and Guan, 2021). Explainability (and broader transparency, such as uncertainty indicators and model limitations) supports safer use by helping users detect implausible outputs, recognize out-of-scope situations, and appropriately calibrate reliance on model recommendations (Nouis et al., 2025).

In pediatric clinical care, the purpose and depth of “explanation” by a clinician is often driven by the audience (e.g., youth and/or caregiver) and clinical context. For children, adolescents, and caregivers, communication on the AI system does not usually require a technical account of model logic, as this may be confusing. Rather, it should provide procedural transparency, including whether an AI tool contributed to a clinical recommendation, what the tool is intended to do (and not do), the level of uncertainty, how data privacy is protected, and how clinicians remain accountable and can override AI outputs (Richter et al., 2025). Professional organizational policies can help shape clinical norms and procurement expectations and therefore serve as influential “soft governance” alongside statutes, regulators, and standards bodies. From an AI governance perspective, transparency includes documenting model purpose, intended users, training data provenance, performance across relevant pediatric subgroups, and known failure modes (Center for Devices, Radiological Health, 2024). Regulatory approaches, such as algorithm change protocols, aim to preserve traceability of adaptive models over time (Gilbert et al., 2021), and post-market monitoring is increasingly emphasized to detect performance drift and safety events (Center for Devices, Radiological Health, 2024). Operationally, transparency should be supported through standardized documentation artifacts (e.g., model cards and datasheets for datasets) and clear communication plans for families when AI meaningfully influences care (Mitchell et al., 2019).

### 3.5 Clinical accountability and integrity

Alongside consent, privacy, equity, and transparency, pediatric AI demands explicit safeguards for how clinicians use and record the technology’s output. Three medico-legal hazards illustrate the need: (i) automation bias, whereby clinicians over-trust a neatly visualized recommendation (Coiera et al., 2018; Khera et al., 2023); (ii) AI-generated documentation that quietly degrades EHR accuracy through factual drift (McCoy et al., 2024); and (iii) the loss of the “whole-child narrative,” such as ambient scribes omitting psychosocial context (Wang et al., 2021; van Buchem et al., 2021). Together, these risks can obscure responsibility and compromise future care or litigation. Clinical accountability would require formal override prompts to counter automation bias, validation checks that synthetic text matches source data, and note templates that preserve family concerns and developmental goals. In doing so, these measures help ensure that both clinical judgment and medico-legal oversight remain with families and their clinical teams.

Economic and environmental sustainability is an increasingly important component of responsible pediatric AI (Nagarajan et al., 2024). Health systems should account for the full lifecycle costs of implementation (integration, monitoring, maintenance, and retraining) and consider how procurement and deployment choices may widen or narrow inequities in access. In parallel, AI models—particularly those requiring substantial computation—can have non-trivial energy and carbon footprints, making efficiency relevant to long-term sustainability (Ueda et al., 2024). Where clinically appropriate, approaches such as model optimization, lightweight deployment strategies, and clear plans for version control, rollback, deprecation, and decommissioning can reduce cost and environmental burden while supporting broader access (Jegham et al., 2025).

## 4 Global perspectives and international guidelines

There have been highlights of key frameworks and reports from global bodies such as UNICEF, the EU, and the WHO, as well as consensus principles emerging from international expert groups. These perspectives often complement U. S. efforts by focusing on broader ethical and human rights issues, particularly in low-resource settings and diverse cultural contexts.

UNICEF’s 2021 AI for Children policy provides a framework based on the UN Convention on the Rights of the Child, outlining nine child-centered requirements for developing AI impacting children (UNICEF, 2021). These include supporting wellbeing, ensuring inclusion and fairness, protecting data and safety, providing transparency, empowering stakeholders, preparing children for AI, and fostering an ethical environment. The guidance emphasizes AI’s role in enhancing children’s development and upholding their rights. UNICEF offers implementation tools and has piloted these guidelines, which serve as a foundation for ethical AI frameworks and influence national strategies. Similarly, the WHO outlined six ethical principles applicable across all ages, emphasizing the need for special care for vulnerable groups, such as children (WHO, 2025). These principles, protecting autonomy, promoting well-being and safety, ensuring transparency, fostering accountability, ensuring inclusiveness and equity, and promoting responsive and sustainable AI, have direct implications for pediatrics. The WHO also cautioned against data biases and commercial exploitation, which could be high-risk concerns relevant to children’s data.

International expert groups are developing consensus frameworks for trustworthy AI in healthcare, such as the Fairness, Universality, Traceability, Usability, Robustness, Explainability (FUTURE-AI framework) (Lekadir et al., 2025). While not pediatric-specific, FUTURE-AI’s principles are universally applicable and address issues such as demographic-based bias, need for audit trails, user-friendliness in pediatric settings, and reliability across pediatric variations. Initiatives such as the UN-led AI for Good (ITU AI for Good, 2020) and WEF’s Responsible AI Playbook (The World Economic Forum, 2025) also contribute to this trend of collaborative global efforts toward safe and ethical AI, recognizing the need for inclusivity and considerations of vulnerable populations.

Global AI regulations and ethical guidelines increasingly emphasize the safety and rights of children, with direct implications for pediatric AI in healthcare. The EU AI Act classifies many healthcare AI applications and systems that use or affect children as high-risk, thereby requiring risk management, transparency, and human oversight (European Parliament, 2023). In the United States, recent federal and state activity has focused on consumer protection and algorithmic discrimination, which can affect both clinical and consumer-facing pediatric AI tools (Mello and Cohen, 2025). Child privacy enforcement has also intensified, including Federal Trade Commission actions that limit monetization and profiling based on children’s data under COPPA (Federal Trade Commission, 2025). Internationally, the OECD AI Principles (OECD, 2025) and the UN Committee on the Rights of the Child highlight child protection in digital environments (OHCHR, 2026), while UNESCO’s recommendation emphasizes human rights, sustainability, and inclusion as cross-cutting obligations (UNESCO, 2022). Together, these developments highlight that regulatory readiness is a moving target and reinforce the need for robust, adaptive governance for pediatric AI deployment.

## 5 The pediatric-AI implementation readiness (PAIR) framework

**Figure 1:**
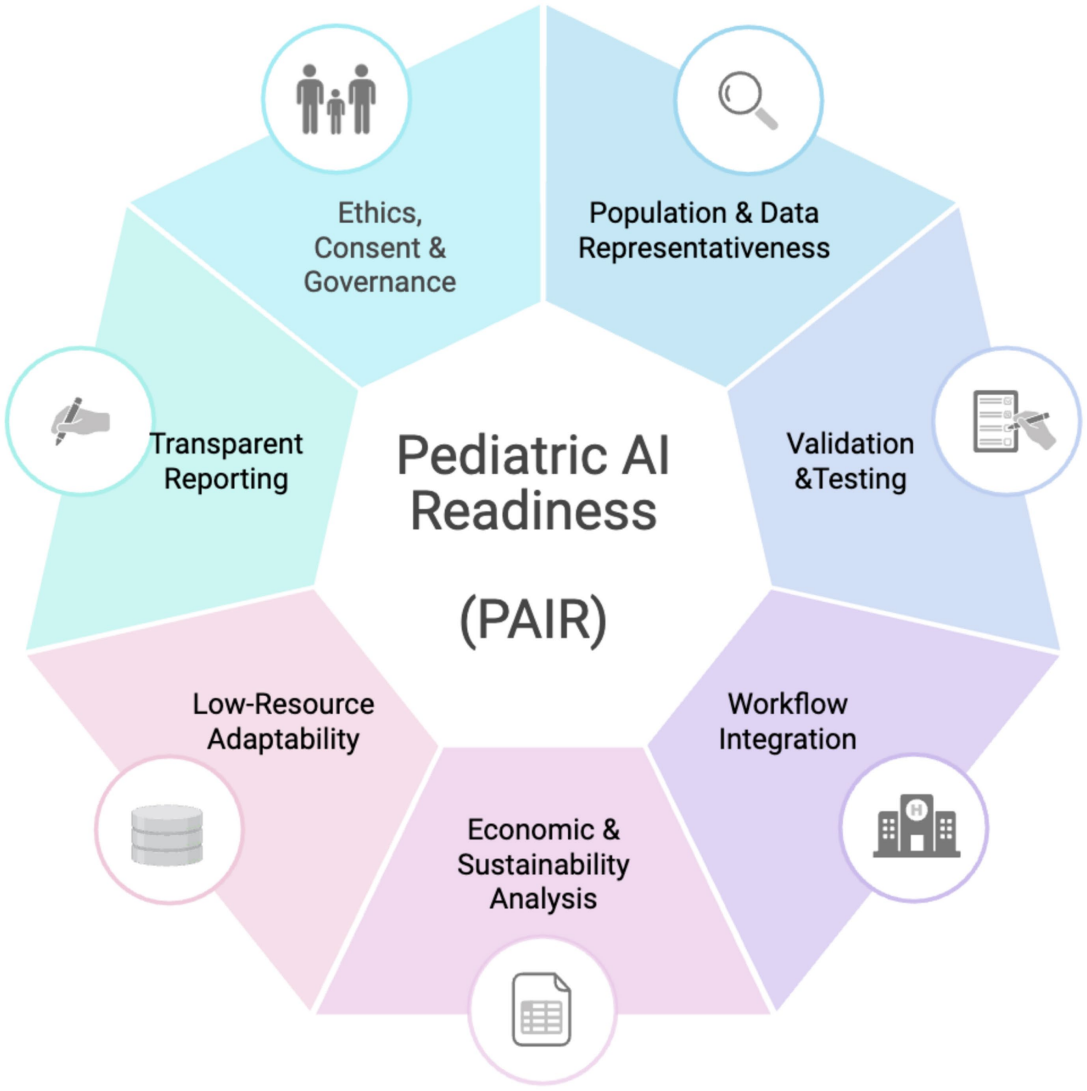
PAIR framework. Created in BioRender. Sezgin (2026).

The preceding sections highlight a critical implementation gap: AI applications in pediatrics are expanding (Section 2), while ethical, data, governance, and workflow challenges unique to children remain unresolved (Sections 3–4). To address this gap, we propose the Pediatric AI Implementation Readiness (PAIR) framework, a pediatric-focused, seven-domain checklist intended to support real-world deployment and scale-up of AI tools in pediatric care. The PAIR is not a reporting guideline and does not prescribe model development methods. Instead, it translates child-specific implementation considerations into operational checkpoints that can be used by investigators, health system leaders, regulators, and funders to assess whether an AI tool is ready to be implemented safely and equitably in a given clinical context (Figure 1).

The evidence synthesized in this review informed the content of each domain, and the domains are organized using implementation science constructs to enhance usability. Specifically, the PAIR aligns with Reach, Effectiveness, Adoption, Implementation, Maintenance (RE-AIM) (Glasgow et al., 2019) and the Consolidated Framework for Implementation Research (CFIR) (Damschroder et al., 2020) constructs, including reach (representative pediatric data), effectiveness (clinical utility and harms), adoption and implementation (workflow fit, oversight, and communication), and maintenance (lifecycle monitoring and sustainability). PAIR complements established guidance that focuses on AI development, evaluation, and reporting (e.g., CONSORT-AI, SPIRIT-AI, TRIPOD-AI, MI-CLAIM, and DEAL) (Liu et al., 2020; Cruz Rivera et al., 2020; Norgeot et al., 2020; Collins et al., 2024; Tripathi et al., 2025) and frameworks addressing governance and deployment (e.g., GMLP, FUTURE-AI, ACCEPT-AI, PEARL-AI, and FAIR-AI) (Chng et al., 2025; U.S. Food and Drug Administration, 2025; Muralidharan et al., 2023; Lekadir et al., 2025; Wells et al., 2025). Its contribution is a pediatric-specific implementation-readiness lens that foregrounds age-stratified validation, adolescent confidentiality, family-centered communication, and context-appropriate equity and sustainability planning (Table 1). The PAIR intentionally avoids prescribing specific model architectures or arbitrary technical constraints; instead, it provides a structured checklist that can be used currently and may be refined into a quantitative rubric as empirical benchmarks emerge. To support pragmatic use, we provide an accompanying self-appraisal checklist with subdomains and example maturity levels (Appendix 1), which institutions can adapt to local workflows and resources.

**Table 1:** PAIR domains and explanations.

Domain	Explanation
1. Ethics, Consent, and Governance	The PAIR operationalizes ethical readiness for real-world pediatric AI use. It focuses on governance and patient-family protections at the point of clinical deployment, not on obtaining individual consent for secondary use of de-identified training data (which is typically governed through institutional review, waivers, and data-use agreements). Readiness includes (i) an AI oversight structure with clinical, data science, ethics, and family/community representation; (ii) documented policies for informing families when AI meaningfully influences care, including developmentally appropriate assent when feasible and longitudinal consent maintenance when services persist over time; (iii) clear accountability and clinician override pathways; and (iv) compliance with relevant privacy and security requirements (e.g., HIPAA and COPPA) and ongoing bias/fairness monitoring.
2. Population and Data Representativeness	Implementation-ready pediatric AI requires data that reflect the population and context in which the tool will be used. Readiness includes the transparent documentation of dataset provenance and limitations; reporting of model performance across pediatric developmental strata relevant to the intended use (e.g., neonatal, infant, child, and adolescent); and disaggregation by key demographic and social determinants where feasible. When pediatric data are sparse, programs should justify mitigation strategies (e.g., multi-site collaboration, federated learning, or carefully validated synthetic augmentation) and explicitly assess generalizability to underrepresented groups.
3. Validation and testing	Validation should demonstrate that performance is adequate for the intended pediatric use case and that risks are understood and mitigated. Readiness includes standardized performance evaluation (e.g., discrimination, calibration, and clinically meaningful utility), age-stratified and subgroup analyses, and external validation across settings. Where feasible, prospective or pragmatic evaluation embedded in workflow should assess safety, workflow impact, and adverse events, with processes for incident review and model update governance. Human factors testing with clinicians and, when relevant, caregivers and adolescents should assess usability, comprehension, and conditions under which users override or over-rely on AI.
4. Workflow Integration	Workflow integration assesses whether the tool can be used safely within pediatric care processes. Readiness includes appropriate EHR and clinical system integration, clear presentation of outputs, alert/notification strategies that minimize fatigue, and audit trails for AI outputs and clinician actions. Pediatric deployment should explicitly address parent-proxy access, adolescent confidentiality, and communication pathways for caregiver-facing interfaces in which AI affects shared decision-making.
5. Economic and Sustainability Analysis	Economic and sustainability readiness evaluate whether the tool can be maintained in routine care. This includes budget impact and cost-effectiveness considerations where appropriate, resource requirements for integration and support, and lifecycle plans for monitoring, retraining, version control, and rollback. Critically, readiness should include explicit deprecation and decommissioning/retirement criteria (e.g., performance drift, guideline changes, equity harms, and vendor changes) and a plan for safe transition to an updated tool or non-AI workflow. Environmental considerations (compute and energy use) may be relevant for high-volume or large-model deployments and can inform choices about optimization and infrastructure.
6. Low-resource Adaptability	Low-resource adaptability considers whether the tool can function across pediatric care settings outside tertiary centers (e.g., community clinics, schools, rural sites, and global health contexts). Readiness may include offline or low-bandwidth functionality, compatibility with commonly available devices, multilingual and health-literacy-appropriate interfaces, and participatory co-design with families and frontline clinicians. Adaptation should be context-dependent and justified by the intended deployment environment rather than imposed as an arbitrary constraint.
7. Transparent Reporting	Transparent reporting supports trust and auditability across development and deployment. Readiness includes clear intended-use statements, documentation of data provenance and evaluation (including subgroup performance), and disclosure of known limitations. Standard documentation artifacts, such as model cards and dataset datasheets, should be used where feasible. For clinical adoption, transparency also includes interpretability or explanation strategies appropriate to the use case, version control and drift monitoring, and traceability of AI-generated outputs (particularly for generative tools).

## 6 Conclusion

Artificial intelligence holds transformative potential to advance pediatric healthcare, enabling earlier disease detection, tailoring therapies to individual physiology, and proactive intervention before crises emerge. Substantial successes already illustrate these possibilities, such as algorithms that read pediatric imaging with expert-level accuracy, predictive models that avert neonatal sepsis, and decision-support systems that shorten diagnostic journeys for rare disorders. However, because children occupy a unique moral and developmental position that necessitates special protections for consent, privacy, and fairness, rigorous safeguards are essential. The full complement of frameworks reviewed in this study converges on a unified imperative: pediatric AI must be safe, effective, fair, transparent, and unequivocally child-centric.

Realization of this vision depends on sustained collaboration among pediatricians, data scientists, ethicists, regulators, families, and patient advocates. Dedicated pediatric standards analogous to those governing drug trials remain urgently needed, particularly to prevent a widening digital divide in low-resource settings. Nevertheless, the trajectory points in a positive direction. By leveraging existing ethical and regulatory guidance and translating it into practical deployment checkpoints, the field can develop AI that augments clinical care while safeguarding children’s rights and dignity. PAIR provides a pragmatic implementation-readiness checklist that can guide planning, evaluation, and responsible scale-up of pediatric AI in real-world settings.

## Data Availability

The original contributions presented in the study are included in the article/Supplementary material, further inquiries can be directed to the corresponding author.
