# Supplementary material for: The pediatric AI readiness framework: bridging evidence to practice in pediatric artificial intelligence
Source: Front Artif Intell. 2026 May 1;9:1800047. doi: 10.3389/frai.2026.1800047 (PMC13176136; doi:10.3389/frai.2026.1800047)
Supplement: Supplementary file 1 [file Data_Sheet_1.PDF]

Appendix 1- Checklist for self appraisal

This is a sample list of items for each of 7 domains:

1. Ethics, Consent & Governance

Subdomain	Description
**1.1 Consent & Assent Processes**	Existence and auditing of processes for obtaining informed consent and developmental assent, with age-appropriate materials and tracking re-consent at defined milestones (e.g., 13, 18).

2. Population & Data Representativeness

Subdomain	Description
**2.1 Age & Developmental Stratification**	Model input/output stratified by pediatric age groups, developmental stages, and adjusted dosing or care recommendations.

3. Validation & Study Design

Subdomain	Description
**3.1 Prospective & Pragmatic Evaluation**	Execution of real-world studies embedded in clinical workflow with active harms monitoring, not just post-hoc analyses.

4. Workflow Integration & Usability

Subdomain	Description
**4.1 EHR & Clinical System Integration**	Seamless bi-directional data exchange with core hospital systems (EHR, PACS, decision support); audit trail preserved.

5. Economic & Sustainability Analysis

Subdomain	Description
**5.1 Cost-Effectiveness & Budget Planning**	Use of budget impact models, QALY estimation, and multi-year financial projections for implementation.

6. Low-Resource Adaptability

Subdomain	Description
**6.1 Offline & Mobile Deployment Capability**	Functionality of AI systems in areas with intermittent internet; ability to operate on low-cost hardware or mobile platforms.

7. Reporting & Open Science

Subdomain	Description
**7.1 Documentation Transparency**	Publication of a model card, training metadata, licensing terms, and intended-use statements.

Example scoring scale:

Subdomain 1.1 – Consent & Assent Processes

Level	Description
**1 – Nascent**	No documented consent or assent process specific to AI use.
**2 – Emerging**	Parental consent used in pilots; child assent optional or ad hoc.
**3 – Aligned**	Structured consent/assent protocols developed; re-consent triggers defined.
**4 – Embedded**	Integrated into EHR workflows; multilingual formats; tracked/audited.

**5 – Leading**	Interactive, age-tailored tools used system-wide; uptake data publicly reported.
